# ER stress in rodent islets of Langerhans is concomitant with obesity and β-cell compensation but not with β-cell dysfunction and diabetes

**DOI:** 10.1038/nutd.2013.35

**Published:** 2013-10-21

**Authors:** O Omikorede, C Qi, T Gorman, P Chapman, A Yu, D M Smith, T P Herbert

**Affiliations:** 1 Department of Cell Physiology and Pharmacology, The Henry Wellcome Building, University of Leicester, University Road, Leicester, UK; 2 Discovery Sciences, High Content Biology, AstraZeneca, Macclesfield, Cheshire, UK; 3 Diabetes & Obesity Drug Discovery, AstraZeneca, Macclesfield, Cheshire, UK; 4 Diabetes & Obesity Drug Discovery, AstraZeneca, Mölndal, Sweden

**Keywords:** ER stress, unfolded protein response, obesity, pancreatic β-cell, type-2 diabetes

## Abstract

**Objective:**

The objective of this study was to determine whether ER stress correlates with β-cell dysfunction in obesity-associated diabetes.

**Methods:**

Quantitative RT-PCR and western blot analysis were used to investigate changes in the expression of markers of ER stress, the unfolded protein response (UPR) and β-cell function in islets isolated from (1) non-diabetic Zucker obese (ZO) and obese female Zucker diabetic fatty (fZDF) rats compared with their lean littermates and from (2) high-fat-diet-fed fZDF rats (HF-fZDF), to induce diabetes, compared with age-matched non-diabetic obese fZDF rats.

**Results:**

Markers of an adaptive ER stress/UPR and β-cell function are elevated in islets isolated from ZO and fZDF rats compared with their lean littermates. In islets isolated from HF-fZDF rats, there was no significant change in the expression of markers of ER stress compared with age matched, obese, non-diabetic fZDF rats.

**Conclusions:**

These results provide evidence that obesity-induced activation of the UPR is an adaptive response for increasing the ER folding capacity to meet the increased demand for insulin. As ER stress is not exacerbated in high-fat-diet-induced diabetes, we suggest that failure of the islet to mount an effective adaptive UPR in response to an additional increase in insulin demand, rather than chronic ER stress, may ultimately lead to β-cell failure and hence diabetes.

## Introduction

Type-2 diabetes mellitus (T2DM) is a disorder that is characterised by high blood glucose concentration in the context of insulin resistance and/or relative insulin deficiency. It causes metabolic changes that lead to the damage and functional impairment of organs and tissues, resulting in increased morbidity and mortality. It is the prevalence of this form of diabetes that is increasing at an alarming rate because of the ‘obesity epidemic', as obesity is a key risk factor in the development of insulin resistance. Although insulin resistance contributes to the pathogenesis of T2DM, it is the failure of pancreatic β-cells to secrete sufficient insulin to maintain normoglycemia, due to a decrease in β-cell function and mass, that results in the development of T2DM (for reviews see:^1, 2, 3^). Yet, the underlying molecular mechanisms that cause the loss of β-cell mass in T2DM are poorly understood. Interestingly, the chronic incubation of isolated islets of Langerhans or clonal β-cell lines with FFAs leads to cell dysfunction and death, which is associated with the induction of ER stress and chronic activation of the unfolded protein response (UPR).^4, 5, 6, 7, 8^ Moreover, markers of ER stress have been found in islets from obese diabetic db/db mice^6^ and in β-cells from non-BMI-matched pancreatic sections from type-2 diabetics compared with non-diabetics.^6^ On the basis of these observations, it has been hypothesised that, in obese individuals, elevated circulatory FFAs act on the β-cell to cause ER stress, resulting in β-cell dysfunction and death.^9^ However, there is little *in vivo* correlative evidence to support this and, indeed, little to no change in the expression of markers of ER stress was observed in BMI-matched non-diabetic obese versus type-2 diabetic obese subjects, although an increase in ER mass was detected in the islets of type-2 diabetic obese subjects.^10^

Zucker Obese (ZO) rats lack a functional leptin receptor, which results in hyperphagia, obesity and insulin resistance.^11^ Yet, these animals remain normoglycemic through a compensatory increase in insulin secretion maintained through increased β-cell mass and possibly function.^12, 13, 14^ Selective inbreeding of glucose-intolerant ZO rats led to the establishment of Zucker diabetic fatty (ZDF) rats.^11^ Male ZDF (mZDF) rats spontaneously develop diabetes between 6 and 12 weeks of age. This is concomitant with a failure of β-cells to adequately adapt to an increase in demand, followed by a decline in β-cell function and mass.^12, 13, 15, 16^ These rats have high levels of circulating FFAs and their islets are susceptible to lipotoxicity.^17^ Therefore, it has been suggested that β-cell dysfunction and death *in vivo* are due to β-cell lipotoxicity.^17^ In contrast, female ZDF (fZDF) rats, although obese and glucose intolerant, are not diabetic because of β-cell compensation.^11, 15^ These animals do not develop diabetes unless they are placed on a high-fat diet (HFD), which results in decreased insulin sensitivity and a failure of the β-cell to adequately compensate, followed by a progressive decrease in β-cell function and mass.^11, 15, 18, 19, 20, 21^ HFD-fed fZDFs (HF-fZDFs) have been proposed to be a good animal model of diet-induced T2DM.^18^ Therefore, using ZO and fZDF rats as models of obesity and HF-fZDF rats as a model of diet-induced diabetes, we investigated whether there is a correlation between ER stress in the islets of Langerhans and (1) obesity and (2) HFD-induced diabetes.

## Methods and procedures

### Chemicals

FCS (foetal calf serum) was purchased from Invitrogen (Life Technologies, Carlsbad, CA, USA). All other chemicals were obtained from Sigma-Aldrich (St Louis, MO, USA) (unless otherwise stated).

### Animal care and dietary treatment

Animals were kept under standard laboratory conditions with free access to food and water. All animals used in these studies were purchased from Charles River Laboratories (Wilmington, MA, USA). Where indicated 12-week-old fZDF were fed either a HFD (60% fat, 20% protein, 20% carbohydrate; D12492; Research Diets, New Brunswick, NJ, USA) or control chow diet (CD, 7.5% fat, 17.5% protein, 75% carbohydrate; RM1, Special Diets Services, Witham, Essex, UK) for 7 weeks.

### Metabolite assays

Tail blood from 16-h-fasted rats was assessed for plasma insulin using an enzyme-linked immunosorbent assay (Ultrasensitive Rat Insulin ELISA kit; Mercodia, Uppsala, Sweden) and glycated haemoglobin (HbA1c) using a Variant II analyser (Bio-Rad, Hercules, CA, USA).

### Islet isolation

Rats were euthanised by CO_2_/O_2_ narcosis and cervical dislocation (without recovery from narcosis). The rats were then weighed and the pancreas was rapidly removed. The islets of Langerhans were isolated as previously described.^22^

### RNA isolation

RNA was extracted as per the manufacturer's instruction (Qiagen RNeasy microkit, Qiagen, Venlo, the Netherlands). The RNA was then DNAse1 (Qiagen)-treated and stored at −80 °C until required. RNA concentration was determined using a Nanodrop ND-1000 spectrophotometer (Thermoscientific, Waltham, MA, USA).

### Gene expression analysis using TaqMan low-density arrays (TLDA)

Predesigned TaqMan primers and probe sets (Table 1) were factory-loaded into the 384 wells of TaqMan low-density arrays (TLDAs; Applied Biosystems, Life Technologies, Carlsbad, CA, USA) as 48 genes per sample with eight samples per card. RNA of 4 μg was complexed to oligo(dT) (0.5 ug ul^−1^) and cDNA was generated using Superscript Reverse Transcriptase III (Invitrogen) as per the manufacturer's instructions. cDNA, equivalent to 110 ng of starting RNA, was mixed with TaqMan Universal PCR Master Mix (Applied Biosystems, Life Technologies) and transferred into a TLDA. Thermal cycling was performed on an Applied Biosystems Prism 7900HT sequence detection system as follows: 2 min at 50 °C, 10 min at 95 °C, 15 s at 95 °C and 1 min at 60 °C for 40 cycles. Expression values were calculated using the comparative C_*T*_ method as previously described.^23^ Expression levels of target genes were normalised to 18S ribosomal RNA. Results were expressed as means±s.e.m.

### Individual TaqMan quantitative RT-PCR

Pre-selected mRNAs were assessed by quantitative RT-PCR using the TaqMan RNA-to-C_*T*_ 1-step kit (Applied Biosystems). Pre-complexed primers were purchased from Applied Biosystems (ABI; Foster City). All samples were run in triplicate on an Applied Biosystems 7900/7700 machine. Expression levels were calculated using the comparative C_T_ method as previously described.^23^ Results were normalised to 18S ribosomal RNA and expressed as fold change.

### MIN6 cell culture and treatment

In this study, Mouse insulinoma-6 (MIN6) cells^24^ were cultured, treated and prepared for SDS-PAGE as previously described.^25^

### SDS-polyacrylamide gel electrophoresis (PAGE) and western blotting

The islets were immediately lysed in 1x Laemmli sample buffer (0.1% 2-Mercaptoethanol, bromophenol blue, 10% Glycerol, 2% SDS, 63 mM Tris-HCl (pH 6.8)), boiled and then stored at −80 °C before western blot analysis. SDS-PAGE and western blotting were performed as described previously.^26^ Anti- phospho-JNK, JNK and rpS6 antibodies were purchased from Cell Signaling Technology (Beverly, MA, USA). Anti-CHOP, ATF4, calreticulin and HYOU1 antibodies were purchased from Santa Cruz Biotechnology (Santa Cruz, CA, USA). Anti-phospho-eIF2α (Ser51), anti-GRP78, anti-phospho-IRE1 and anti-GRP98 antibodies were purchased from Biosource (Camarillo, CA, USA), BD-signal transduction (Sparks, MD, USA), Novus Biologicals (Cambridge, UK) and Abcam (Cambridge, UK), respectively. Detection was by horseradish peroxidase-linked secondary antibodies and enhanced chemiluminescence (Cheshire Sciences Ltd, Cheshire, UK).

### Statistical analysis

Comparisons of more than two groups were made using two-way analysis of variance. A difference was defined as significant when *P*<0.05.

## Results

### ER stress in the islets of Langerhans isolated from Zucker obese rats

**Figure 1:**
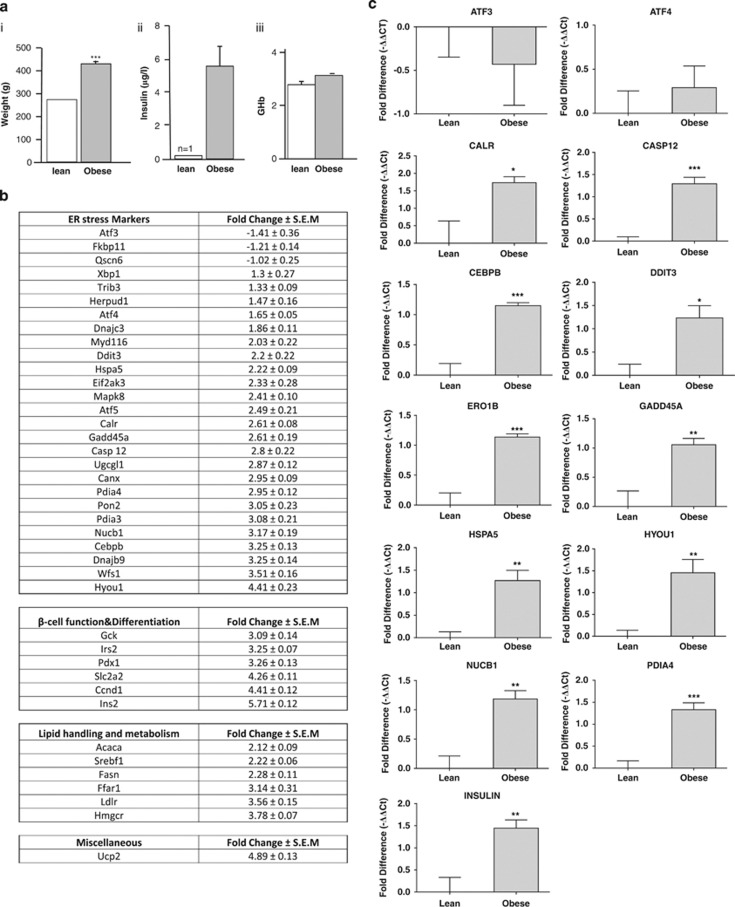
Obesity leads to increased ER stress in ZO rats. (**a**i) Body weight in grams, (**a**ii) plasma insulin after an overnight fast and (**a**iii) % GHb in age-matched Zucker rats (obese) and their lean littermates (lean). (**b**) Differential gene expression between Zucker rats (obese) and their lean littermates (lean) assessed using TLDA is expressed as a fold change and normalised to 18S ribosomal RNA. Data are shown as mean±s.e.m. (Zucker obese, *n*=5; lean control, *n*=4). (**c**) Relative expression of selected transcripts from the islets of Zucker rats (obese) and their lean littermates (lean) assessed by single-gene Taqman RT-qPCR. Results are expressed as relative expression levels and normalised to the housekeeping gene ribosomal protein P2 (RPP2). Values are mean±s.e.m. determined from control lean (*n*=4) and obese rats (*n*=5). Statistical significance was determined using an unpaired two-tailed Student *t*-test. *P<0.05; **P<0.01; ***P<0.001 obese versus lean control for each gene.

To determine whether obesity is associated with ER stress and the induction of the UPR in β-cells, islets of Langerhans were isolated from 11-week-old male Zucker Obese (ZO) rats *(fa/fa)* and their age-matched lean littermates *(Fa/fa)*, and differences in the expression of 41 selected mRNAs (see Table 1), including 27 markers of ER stress, markers of β-cell function, differentiation and lipid handling, were determined using Taqman Low-Density Arrays (TLDAs). The 11-week-old ZO rats used in this study weighed significantly more than their lean littermates fed on the same standard chow diet (Figure 1ai). In addition, they were hyperinsulinemic but maintained fairly normal glycemic control (Figures 1aii and aiii). The expression of 21 out of 27 markers of ER stress (that is, 78%) increased by 1.5X or more in the islets of ZO rats compared with their lean littermates. These included mRNAs encoding chaperone proteins, quality control proteins and foldases, such as hypoxia-upregulated 1 (*Hyou1*), protein disulphide isomerase family A member 4 (*Pdia4*), calreticulin (*Calr*), heat shock 70 kDa protein 5 a.k.a BiP (*Hspa5*), protein disulphide isomerase family A member 3 (*Pdia3*) and calnexin (*Canx*). Other characteristic markers of ER stress were also upregulated, including activating transcription factor 4 (*Atf4*), DNA damage-inducible transcript-3/C/EBP homologous protein (*Ddit3/Chop*), and growth arrest and DNA damage-inducible gene 34 (*Gadd34/Myd116*). Significant increases in the expression of markers of β-cell function, such as insulin-2 (*Ins2*), were also detected in the islets of ZO rats, indicative of β-cell compensation. There was also evidence of increased lipid handling and metabolism in the islets of ZO rats as the expression levels of HMG-CoA reductase (*Hmgcr*), sterol regulatory element binding protein-1 (*Srebf1*), low-density lipoprotein receptor (*Ldlr*) and fatty acid synthase (*Fasn*) were all significantly increased (Figure 1b).

To confirm the differences in the expression of markers of ER stress in the islets of ZO versus lean rats obtained using TLDA, a more stringent assessment of changes in the mRNA expression was made using single-gene RT-qPCR. In agreement with the TLDA data, the expression levels of the ER stress markers calreticulin, *Hspa5* (BiP), *Hyou1/Orp150*, *Pdia4*, growth arrest and DNA damage-inducible 45α (*Gadd45A*), *Ddit3/Chop*, caspase-12 (*Casp12*) and nucleobindin (*Nucb1*) were all significantly upregulated in the islets of ZO rats compared with lean control rats (Figure 1c). Moreover, the expression of an additional ER stress marker, ER-oxidoreductin 1β (*Ero1β*), was upregulated in the islets of ZO rats compared with lean control rats (Figure 1c). However, in contrast to the TLDA data, no significant increase in the mRNA expression of *Atf4* was detected (Figure 1c). Nonetheless, taken together, the data provide evidence that the islets from ZO rat have undergone an adaptive UPR, which is indicative of previous, and possibly continued, exposure to increased levels of ER stress.

### ER stress in the islets of Langerhans isolated from female Zucker diabetic female (fZDF) rats

**Figure 2:**
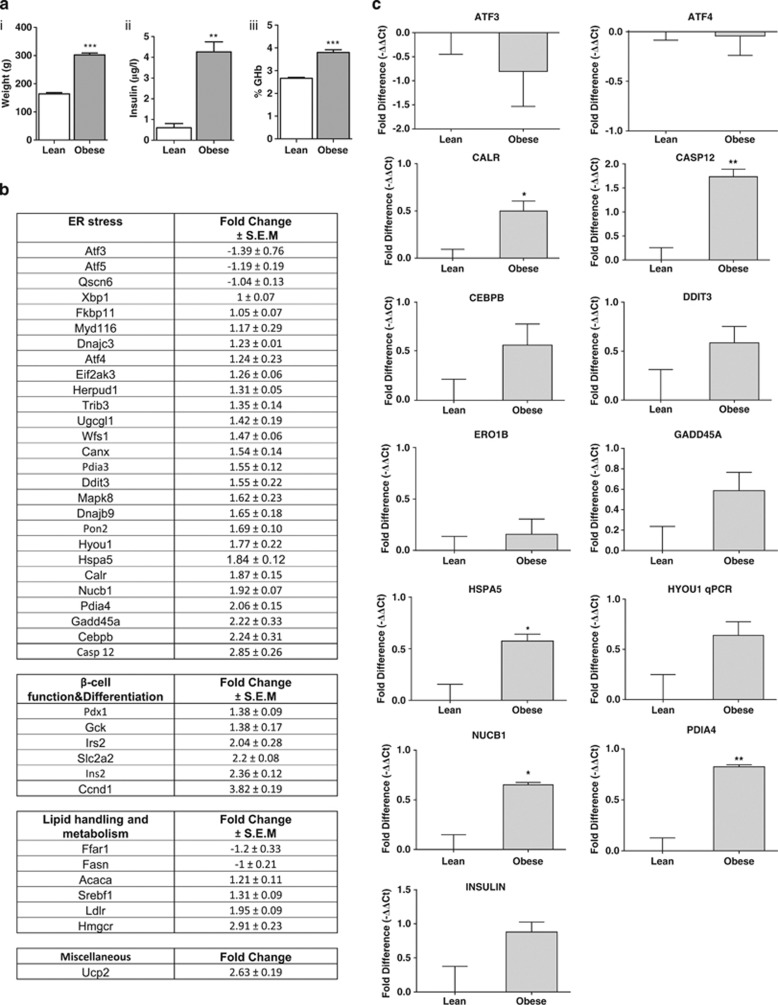
Obesity leads to increased ER stress in female ZDF rats. (**a**i) Body weight in grams, (**a**ii) plasma insulin after an overnight fast and (**a**iii) %GHb in fZDF rats (obese) and their lean littermates (lean). (**b**) Differential gene expression in the islets of fZDF rats (obese) and their lean littermates (lean) assessed using TLDA is expressed as a fold change and is normalised to 18S ribosomal RNA. Data are shown as mean±s.e.m. (*n*=3). (**c**) Relative expression of selected transcripts from the islets of fZDF rats (obese) and their lean littermates (lean) by single-gene Taqman RT-qPCR. Results are expressed as relative expression levels and normalised to ribosomal protein P2 (RPP2). Values are mean±s.e.m. determined from control lean (*n*=3) and obese rats (*n*=3). Statistical significance was determined using an unpaired two-tailed Student *t*-test. *P<0.05; **P<0.01; ***P<0.001 obese versus lean control for each gene.

To investigate whether obesity also correlates with the induction of UPR in islets from an alternative rodent model of obesity, expression of key markers of ER stress were assessed in obese female Zucker diabetic fatty (fZDF) rats *(fa/fa)* and their heterozygous lean littermates *(Fa/fa)*. Unlike male ZDF rats, fZDF rats do not spontaneously develop diabetes and maintain normoglycemia despite insulin resistance through compensatory increases in insulin secretion.^18^ The 12-week-old fZDF rats used in this study weighed significantly more than their age-matched lean littermates, maintained good glycemic control and were characteristically hyperinsulinemic (Figure 2a).

Using TLDAs (list of genes shown in Table 1), it was found that the mRNA expression of nearly all markers of ER stress analysed increased in the islets of fZDF rats in comparison with their lean littermates. Of these, the expression of 52% increased by more than 1.5-fold or more. These included *Pdia4*, *Calr*, *Hspa5 (BiP)*, *Pdia3* and *Ddit3* (CHOP) mRNA (Figure 2b). In addition, there were significant increases in the mRNA expression of insulin 2 (*Ins2*), insulin receptor substrate (*Irs2*) and glucose transporter-2 (*Glut2/Slc2a2*), markers of β-cell function and differentiation, and *Srebf1*, *Ldlr* and *Hmgcr*, markers of lipid handling and metabolism (Figure 2b).

To confirm the results obtained using TLDA, the expression of a selected set of genes encoding markers of ER stress was analysed by single-gene RT-qPCR (Figure 2c). The expression levels of mRNAs encoding *Calr*, *Hspa5*, *Pdia4* and *Nucb1* were all found to be significantly upregulated in the islets of fZDF rats in comparison with lean controls, indicative of an adaptive UPR (Figure 2c). Although there was a trend towards an increase in the expression of mRNA encoding other markers of ER stress (that is, *caspase-12*, *Hyou1*, *Gadd45A*, *Ddit3/Chop* and *C/ebpβ*), these differences proved statistically insignificant (Figure 2c). In addition, there were no differences in the expression of *Ero1β*, *Atf3* and *Atf4* (Figure 2c). Nonetheless, taken together, these results provide evidence that a subset of mRNA, reported to be upregulated in response to ER stress, is upregulated in the islets of fZDF rats compared with their lean littermates.

### ER stress in the islets of Langerhans isolated from age-matched fZDF rats compared with high-fat-fed fZDF (HF-fZDF) rats

**Figure 3:**
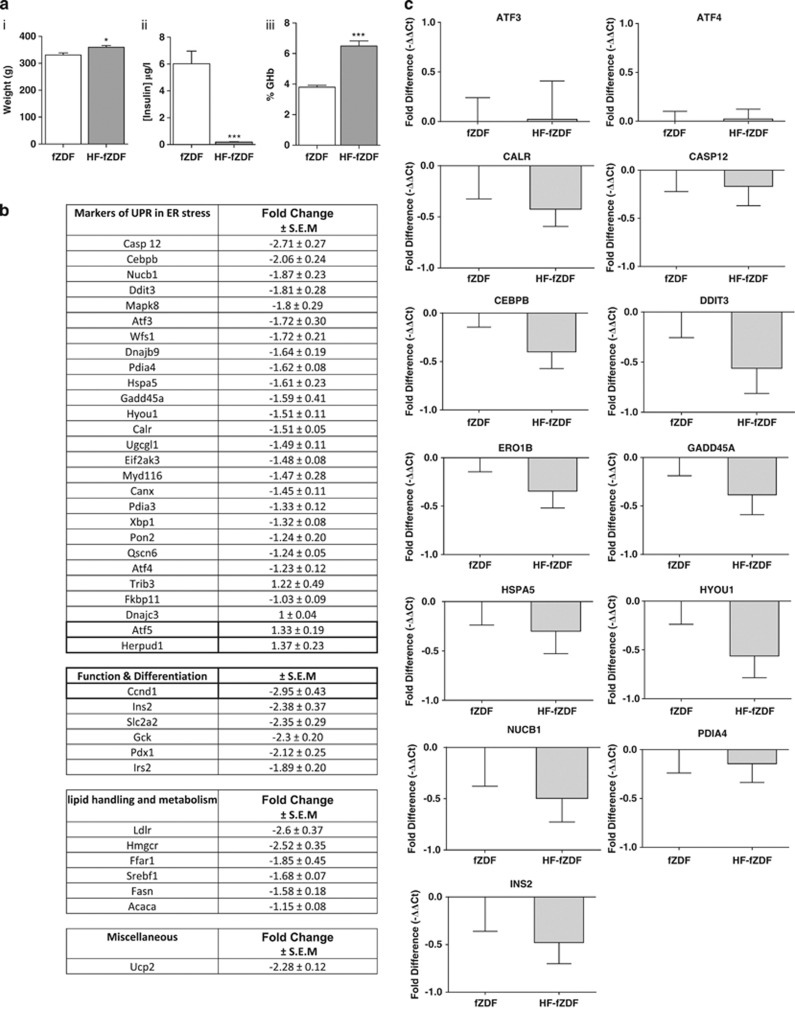
Obesity-induced ER stress in the islets of Langerhans is not exacerbated by diet-induced diabetes. (**a**i) Body weight in grams, (**a**ii) plasma insulin after an overnight fast and (**a**iii) %GHb in chow-fed fZDF rats and HF-fZDF rats. (**b**) Differential gene expression in the islets of fZDF rats and HF-fZDF rats assessed using TLDA and expressed as a fold change and normalised to 18S ribosomal RNA. Data are shown as mean±s.e.m. (fZDF, *n*=3; HF-fZDF, *n*=3). (**c**) Relative expression of selected transcripts from the islets of HF-fZDF versus chow-fed fZDF rats by single-gene Taqman RT-qPCR. Results are expressed as relative expression levels and normalised to ribosomal protein P2 (RPP2). Values are mean±s.e.m. determined from control chow-fed fZDF (*n*=6) and HF-fZDF rats (*n*=6). Statistical significance was determined using an unpaired two-tailed Student *t*-test. No significant changes were found.

To investigate whether there is a correlation between the development of obesity-associated diabetes and ER stress signalling, fZDF rats were placed on a high-fat diet (HFD) for 7 weeks to induce diabetes. This has been shown to correlate with a decrease in β-cell function.^18^ HFD-fed fZDF rats (HF-fZDF) weighed significantly more than their obese littermates fed on a standard chow diet for the same duration (Figure 3ai). These rats also had reduced fasting plasma insulin concentrations (Figure 3aii), suggestive of β-cell dysfunction, and increased glycohemoglobin (GHb) (Figure 3aiii), indicative of chronic hyperglycemia.

RNA was extracted from the islets of Langerhans isolated from these rats and the comparative expression levels of markers of ER stress between fZDF and the HF-fZDF rats were assessed using TLDAs (see Table 1). The expression of all the markers of ER stress either decreased or was unchanged in the islets isolated from HF-fZDF compared with the chow-fed fZDF (Figure 3b). Markers of β-cell function and differentiation, including the expression of insulin-2 (*Ins2*), Glut2 (*Slc2a2*), glucokinase (*Gck*) and *Pdx1*, were significantly decreased in the islets of the HF-fZDF rats in comparison with chow-fed control fZDF rat islets (Figure 3b), suggestive of a decrease in β-cell function and indicative of β-cell de-differentiation. Surprisingly, there were also significant decreases in the expression of mRNAs involved in lipid handling and metabolism, including *Srebf1* and *Ldlr* (Figure 3b). The expression of *Ucp2*, which has been reported to be upregulated upon exposure to FFAs,^27^ was also significantly reduced in the islets of the HF-fZDF rats compared with chow-fed fZDF control rats (Figure 3b).

**Figure 4:**
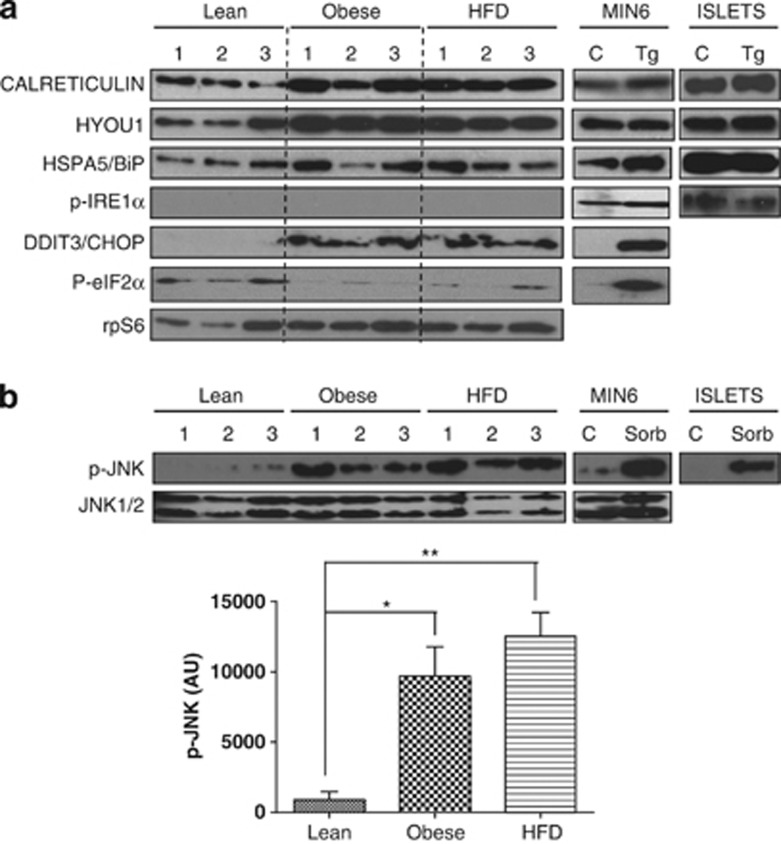
Protein expression of downstream markers of ER stress in islets from lean, obese and high-fat-diet-fed fZDF rats. Protein lysates from islets isolated from three age-matched fZDF rats (obese), HF-fZDF (HFD) rats and their heterozygous lean littermates (lean) were separated by SDS-PAGE and analysed by western blotting. As controls, lysates from the islets isolated from Wistar rats or MIN6 cells treated with 1 μM thapsigargin for 2 h or sorbitol for 1 h were run alongside. Proteins were detected using antisera against (**a**) Calreticulin, HYOU1, GRP78, p-IRE1α, p-eIF2α and CHOP and as protein loading control rpS6 and (**b**) p-JNK and total JNK1/2 as loading control. Western blots were quantified using Image-J software.

To confirm the results obtained using TLDA, the expression of a selected set of mRNAs encoding markers of ER stress was analysed by single-gene RT-qPCR (Figure 3c). No significant changes in the expression of these selected markers of ER stress were detected, including *Hyou1*, *Hspa5* (BIP) or *Ddit3* (CHOP). Changes in the expression of insulin mRNA were also investigated. Although there was a trend towards a decrease in the expression of insulin, possibly mediated though a decrease in β-cell number per islet, these changes proved insignificant. Moreover, normalisation of the expression of markers of ER stress using insulin as the reference gene also revealed no significant increase in the expression of markers of ER stress in HFD-fed animals (results not shown). This is of importance as it provides evidence that potential changes in β-cell number per islet through increased rates of apoptosis and/or dedifferentiation are unlikely to have had any significant effect on the results presented. Taken together, these results indicate that, in this model of diabetes, there are no increases in the mRNA expression of markers of ER stress in the islets of diabetic versus non-diabetic animals. However, changes in mRNA expression do not necessarily reflect changes in protein expression. Therefore, changes in protein expression of selected markers of ER stress in the islets isolated from three age-matched, lean *(Fa/fa)*, fZDF (obese) and HD-fZDF (obese diabetic) rats were determined by Western blot analysis. The expression levels of calreticulin, HSPA5/BIP, HYOU1 and CHOP were all increased in the islets of fZDF rats compared with their lean control (Figure 4a), thus generally reflecting differences in mRNA expression determined by TLDA and single-gene RT-qPCR analysis (Figures 2b and c). Yet, no difference in the expression of these markers of ER stress was detected in the islets of HF-fZDF rats compared with fZDF rats (Figure 4a), thus confirming the mRNA expression data (Figures 3b and c). These data provide evidence that, in this model of diabetes, there are no increases in the expression of markers of ER stress in the islets from age-matched obese diabetic versus obese non-diabetic animals. CHOP expression can be upregulated through an increase in PERK-dependent phosphorylation of eukaryotic initiation factor 2α (eIF2α). However, no significant changes in eIF2α phosphorylation were detected between the groups (Figure 4a).

The phosphorylation status of c-jun N-terminal kinase (JNK), a marker of cellular stress, was also examined by western blot analysis. A significant increase in the phosphorylation state of JNK was observed in the islets of fZDF rats in comparison with lean controls (Figure 4b). However, no statistically significant increase in the phosphorylation of JNK was observed in the islets of HF-fZDF rats in comparison with fZDF (Figure 4b). JNK phosphorylation can be activated by the chronic activation of IRE1.^28^ However, no phosphorylation of IRE1 (an indicator of its activity) was detected in the ZDF islets, although IRE1 phosphorylation was detected in both control and thapsigargin-treated Wistar rat islets (Figure 4a).

## Discussion

Zucker Obese (ZO) and obese fZDF rats maintain glucose homeostasis despite severe insulin resistance through an adaptive compensation in β-cell mass and function.^12, 13, 14, 15, 19, 20, 29, 30, 31^ This is supported by the results presented in this report showing that the expression levels of mRNAs implicated in β-cell function, growth and differentiation, such as *Pdx1*, *Ccnd1* (cyclin-D1), *Slc2a2* (Glut2) and *Gck* (glucokinase), are increased in the islets of these rodent models of obesity (Figures 1 and 2), and, although we have not examined β-cell function or growth directly in this study, many other investigators have. For example, Goh *et al.* demonstrated, using the two-step hyperglycaemic clamp method, that basal insulin secretion is elevated but glucose-stimulated insulin secretion (GSIS) *in vivo* is normal in both obese Zucker and female ZDF rats (fZDF).^32^ Moreover, a number of groups have reported adaptations in key metabolic pathways in Zucker rats.^29, 30, 31^ In addition, increased β-cell mass in these animal models has been shown to be mediated, at least in part, by an increase in β-cell number and size.^13, 14, 19, 20^

**Figure 5:**
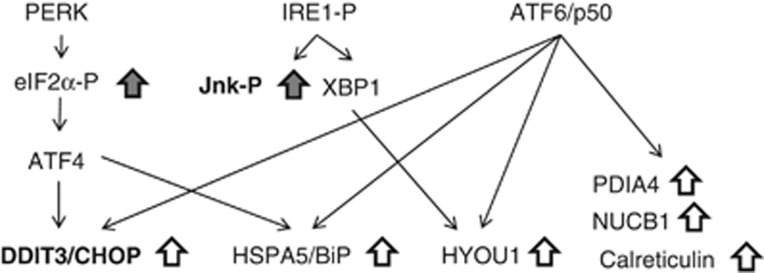
Simplified schematic figure showing the relationship between upstream transducers of the UPR and selected downstream targets. Open arrows represent the direction of change in the expression of selected mRNA/protein in fZDF obese rats compared with their lean controls. Shaded arrows represent the direction of change in the phosphorylation of selected proteins in fZDF obese rats compared with their lean controls. Pro-apoptotic genes are represented in bold.

Importantly, in this study we show that there is an increase in the expression of markers of ER stress, including the ER chaperones and foldases *Hyou1*, *Hspa5* (BiP), *Pdia4*, *Calr* (calreticulin) and *Calx* (calnexin) (Figures 1, 2, 4 and 5). These proteins are important in increasing protein folding capacity in the ER, and thus the upregulation of their expression is likely an important compensatory adaptation to facilitate the increase in insulin synthesis required to maintain enhanced β-cell function. In accordance with this, the obese rats in this study were significantly hyperinsulinemic. Although obesity led to an increase in many markers of ER stress, there was little evidence to indicate sustained activation of the UPR in these islets. For example, we could not detect any increase in the phosphorylation of IRE1 and eIF2α (Figure 4), nor could we detect any significant increase in the expression of *Atf4* and *Xbp1* (Figures 1 and 2), proximal targets of PERK and IRE1, respectively^9^ (Figure 5). Yet, the phosphorylation of JNK (Figure 4), a kinase that can be activated by chronic IRE1 activation,^28^ is elevated in the islets of these obese animals, as is the expression of CHOP (Figure 4), a potentially pro-apoptotic protein that can be upregulated through both the IRE1 and PERK arms of the UPR^33, 34^ (Figure 5). Increased CHOP expression and JNK phosphorylation is often provided as evidence for chronic ER stress. However, both of these proteins can be upregulated/activated in response to a diverse range of stresses including oxidative and inflammatory stress,^35, 36, 37, 38^ which have been reported to occur in and contribute to β-cell dysfunction and death in the development of T2DM (for review, see Poitout and Robertson^39^). Regardless of whether these changes in CHOP expression and JNK phosphorylation are, or are not, a result of ER stress, the islets are clearly stressed but this stress is not, in and of itself, sufficient to lead to the development of β-cell dysfunction and apoptosis in the islets of these obese rodents. Taken together, we conclude that, with the development of obesity and an increase in insulin demand, the β-cells experience ER stress, resulting in an adaptive UPR that is necessary to effectively sustain the increase in insulin demand brought about by both insulin resistance and increased body mass. Although the islets from these obese animals show signs of stress, the source of this stress is far from clear and, apparently, does not adversely affect β-cell function. Interestingly, Chan *et al.* recently reported that β-cell compensation in obese mice also correlates with an increase in the expression of markers of an adaptive UPR.^40^

fZDF rats, fed a high-fat diet (HF-fZDF), develop type-2 diabetes. This has been shown to be concomitant with failure of the β-cells to adequately compensate, followed by a decrease in β-cell function and mass manifested by a progressive decline in plasma insulin levels accompanied by a rise in plasma glucose concentration.^19, 20, 21, 32, 41^ In agreement with these previous studies, we also show that HF-fZDF animals have decreased plasma insulin.^18, 19, 20^ In addition, we show that these animals have decreased insulin expression and a decrease in the expression of markers of β-cell function and differentiation, suggestive of a decline in β-cell function (Figure 3b). This is consistent with studies demonstrating the loss of GSIS and a drop in the disposition index in HF-fZDF rats.^32, 41^ Previous reports have suggested that progressive β-cell dysfunction in models of type-2 diabetes is caused, at least in part, through chronic ER stress, possibly as a result of elevated levels of circulating FFAs and glucose.^42^ However, compared with obese, chow-fed and age-matched fZDF rats, metabolic changes in the HF-fZDF were not accompanied by an increase in the expression of markers of ER stress/UPR (Figures 3 and 4). Importantly, these results are in agreement with microarray data from human subjects, which showed little to no changes in the expression of markers of ER stress in β-cell-enriched samples isolated from BMI-matched obese non-diabetic versus obese type-2 diabetic subjects.^10^ Moreover, we showed that there was no potentiation in the expression of the pro-apoptotic proteins CHOP and caspase-12 or any increase in the phosphorylation of JNK (Figures 3 and 4). However, it is possible that there is increased nuclear localisation of CHOP mediated, for example, through an increase in liver inhibitory protein (LIP) expression, an isoform of C/EBPβ.^43^ These observations indicate that, at least in this rodent model of HFD-induced diabetes, increased obesity, insulin resistance and insulin demand fail to increase ER stress and/or further activate the UPR. This conclusion is supported by recently published findings showing that the progression of β-cell dysfunction in diabetes prone db/db mice does not correlate with an increase in the expression of either ATF4 or CHOP.^40^

Interestingly, the culturing of ‘compensated' islets in FFA isolated from pre-diabetic mZDF rats resulted in a decrease in β-cell function and a reduction in pre-existing compensatory changes that had occurred *in vivo*.^44^ It is therefore possible that the development of β-cell dysfunction and apoptosis in obesity-associated type-2 diabetes occurs as a result of a failure of the UPR to further adapt to increased demand as opposed to the chronic activation of UPR signalling. In support of this, BiP overexpression in β-cells protects mice from high-fat-diet-induced diabetes.^45^ This report should therefore prompt a re-evaluation of the role of ER stress in islet dysfunction and the potential use of pharmacological inhibitors of the UPR in the treatment of type-2 diabetes.

## Figures and Tables

**Table 1:** List of genes on the Taqman low-density array (TLDA) whose mRNA expression was determined by qPCR

*Markers of ER stress*	*Gene*	*Aliases*	*Ref seq*	*Rat assay no*
Activating transcription factor 3	Atf3		NM012912.1	Rn00563784_m1
Activating transcription factor 4	Atf4	CREBP2	NM024403.1	Rn00824644_g1
Activating transcription factor 5	Atf5	Atfx	NM172336.3	Rn00597319_m1
CCAAT/Enhancer binding protein, beta	C/EBPβ	LAP/TF5/NF-IL6	NM024125.4	Rn00824635_s1
Calreticulin	Calr		NM022399.2	Rn00574451_m1
Calnexin	Canx		NM172008.2	Rn00596877_m1
Caspase 12	Casp 12			Rn00590440_m1
Growth arrest and DNA damage-inducible protein	Ddit3	GADD153/CHOP(10)	NM024134	Rn00492098_g1
DnaJ (Hsp40) homologue, subfamily B, member 9	Dnajb9	ERdj4	NM012699.2	Rn00562259_m1
DnaJ (Hsp40) homologue, subfamily C, member 3	Dnajc3	HSP40, DnaJ, p58	NM022232.1	Rn00573712_m1
PRKR-like endoplasmic reticulum kinase	Eif2ak3	PERK/PEK	NM031599.1	Rn00581002_m1
FK506 binding protein 11	FkBP11	FkBP19	NM001013105.1	Rn01532810_m1
Growth arrest and DNA damage-inducible, alpha	Gadd45a	DDIT1	NM024127.2	Rn00577049_m1
Homocysteine-inducible, ER stress-inducible	Herpud1	Herp, Mif1	NM053523.1	Rn00585371_m1
Heat shock 70 kDa protein 5	Hspa5	BiP/GRP78	NM013083.1	Rn00565250_m1
Hypoxia upregulated 1	Hyou1	orp150/Cab140	NM138867.2	Rn00593982_m1
Mitogen-activated protein kinase 8	Mapk8	JNK	XM341399.3	Rn01453358_m1
Myeloid differentiation primary response gene 116	Myd116	GADD34	NM133546.2	Rn00591894_m1
Nucleobindin	Nucb1		NM053463.1	Rn00584973_m1
Protein disulphide isomerase family A, member 3	Pdia3	ERp57/GRP58	NM017319	Rn00569027_m1
Protein disulphide isomerase family A, member 4	Pdia4	ERp72/ERP70	NM053849.1	Rn00587766_m1
Paraoxonase 2	Pon2		NM001013082.1	Rn01456019_m1
Quiescin Q6 sulfhydryl oxidase 1	Qscn6	qsox1	NM053431.3	Rn00584808_m1
Tribbles 3	Trib3	SINK/SKIP3	NM144755.2	Rn00595314_m1
UDP-glucose ceramide glucosyltransferase-like 1	Ugcgl1	UGGT/UGTR	NM133596.1	Rn00592293_m1
Wolframin	Wfs1	DFNA14	NM031823.1	Rn00582735_m1
X-box binding protein 1	Xbp1	TREB5	NM001004210.1	Rn01752572_g1
				
*Markers of β-cell function and differentiation*	*Gene*	*Aliases*	*Ref seq*	*Rat assay no*
Cyclin D1	Ccnd1	PRAD1	NM007631	Rn00432359_m1
Glucokinase/ Hexokinase 4	Gck	MODY2/HK4	NM010292	Rn00561265_m1
Insulin 2	Ins2		NM008387	Rn01774648_g1
Insulin receptor substrate 2	Irs2		NM001168633.1	Rn01482270_s1
Pancreatic and duodenal homeobox 1	Pdx1	MODY4	NM008814	Rn00755591_m1
Glucose transporter type 2	Slc2a2	GLUT2	X78722	Rn00563565_m1
				
*Markers of lipid handling and metabolism*	*Gene*	*Aliases*	*Ref seq*	*Rat assay no*
Acetyl CoA carboxylase	Acaca	ACCA	AAG01858	Rn00573474_m1
Fatty acid synthase	Fasn	Fas	NM007988	Rn00569117_m1
Free fatty acid receptor 1	Ffar1	GPR40	NM153304.1	Rn00824686_s1
HMG-CoA reductase	Hmgcr		M62766	Rn00565598_m1
Low density lipoprotein receptor	Ldlr	FHC	NM010700	Rn00598438_m1
Sterol regulatory element binding protein–1	Srebp1	ADD1	XM213329.4	Rn01495763_g1
				
*Miscellaneous*	*Gene*	*Aliases*	*Ref seq*	*Rat assay no*
Uncoupling protein 2	Ucp2	SLC25A8	U69135	Rn01754856_m1

## Notes

Dr O Omikorede, Dr TP Herbert and Cheng Qi have nothing to disclose, whereas Dr T Gorman, P Chapman, A Yu and DM Smith are employees and shareholders of AstraZeneca Plc.
